# Decoding the bonding in Nd_2_Fe_14_B: a synchrotron charge density perspective on a rare-earth magnet

**DOI:** 10.1107/S2052252525008917

**Published:** 2025-10-27

**Authors:** Yu-Sheng Chen

**Affiliations:** a University of Chicago Pritzker School of Molecular Engineering, NSF’s ChemMatCARS 9700 South Cass Ave., Building 434D Lemont IL 60439 USA

**Keywords:** rare-earth magnets, bonding, charge density, high-energy X-ray diffraction

## Abstract

High-energy synchrotron X-ray diffraction reveals the bonding topology and charge distribution in Nd_2_Fe_14_B, clarifying the atomic-scale origins of its exceptional magnetic performance. The study by Vosegaard *et al.* [(2025). *IUCrJ***12**, 658–669] provides the first detailed charge density analysis of a heavy-rare-earth magnet, guiding future magnet design.

Neodymium–iron–boron, Nd_2_Fe_14_B, is a material of both technological significance and scientific complexity. Its importance stems from being the critical phase behind the strongest known permanent magnet, discovered in 1982, which revolutionized magnet research (Croat *et al.*, 1984 ▸; Sagawa *et al.*, 1984 ▸) and continues to underpin applications from wind turbines to electric vehicles, making it essential for the transition to greener forms of energy.

The tetragonal crystal structure of Nd_2_Fe_14_B, stabilized by boron, was determined by Shoemaker *et al.* (1984 ▸) and provides exceptionally high magnetocrystalline anisotropy from neodymium and large magnetic moments from iron, resulting in energy products significantly exceeding those of ferrite and AlNiCo magnets. This combination of properties enables smaller, lighter and more efficient devices, making Nd_2_Fe_14_B magnets indispensable in technologies such as electric vehicle motors, wind turbines, hard-disk drives, medical imaging equipment and consumer electronics. Despite these advantages, the compound has limitations, including sensitivity to high temperatures and susceptibility to corrosion, which often necessitate the addition of rare-earth elements like dysprosium or protective coatings to enhance durability. Overall, Nd_2_Fe_14_B is vital for advancing modern technology due to its unique balance of structural stability, magnetic strength and wide-ranging applications.

Although Nd_2_Fe_14_B is widely used, its fundamental chemical bonding and electronic structure remained poorly understood due to the complexity of its crystal structure (with 68 open-shell atoms per unit cell) and the difficulty of probing valence electron interactions in heavy-element compounds (Gu & Ching, 1987 ▸; Morishita *et al.*, 2020 ▸). In the current issue of *IUCrJ*, Vosegaard *et al.* (2025 ▸) use high-energy synchrotron single-crystal X-ray diffraction to measure diffraction intensities suitable for multipole modeling of the electron density with the aim of resolving this knowledge gap. To achieve this, they collected data at a low temperature, 25 K, using a very short wavelength of only 0.2482 Å to minimize absorption and extinction effects. The modeling successfully reveals details such as local anisotropy in Fe bonding, charge distribution among Nd, Fe and B, and the crucial role of a single Fe atom in stabilizing the 3D magnetic framework. Their study determines bond topologies, atomic charge distributions (Bader charges), *d*-orbital electron populations and Debye temperatures, thereby linking the local chemical environment to the material’s extraordinary magnetism. This represents the first X-ray charge density analysis of a heavy-atom rare-earth magnet, addressing the ‘complex atomic structure fundamentally responsible for the outstanding properties’ of Nd_2_Fe_14_B.

In summary, the article demonstrates that Nd_2_Fe_14_B is important not only for its applications in modern technology and sustainability, but also as a benchmark system in materials science. Understanding its bonding and electronic structure offers deeper insight into the atomic-scale mechanisms behind its exceptional magnetic properties, with implications for future magnet design and materials discovery.

[The thumbnail image for this article, showing the structure of Nd_2_Fe_14_B viewed down the *a* axis, is reproduced from Vosegaard *et al.* (2025 ▸).]
